# Analysis of miRNAs in milk of four livestock species

**DOI:** 10.1186/s12864-024-10783-4

**Published:** 2024-09-14

**Authors:** Filippo Cendron, Umberto Rosani, Marco Franzoi, Carlo Boselli, Flavio Maggi, Massimo De Marchi, Mauro Penasa

**Affiliations:** 1 Department of Agronomy, Food, Natural Resources, Animals and Environment (DAFNAE), University of Padova, Viale Dell’Università 16, Legnaro (PD), 35020 Italy; 2 Department of Biology (DiBio), University of Padova, Viale Giuseppe Colombo 3, Padua, 35131 Italy; 3 Istituto Zooprofilattico Sperimentale del Lazio E Della Toscana “M. Aleandri” - National Reference Centre for Ovine and Caprine Milk and Dairy Products Quality (C.Re.L.D.O.C.), Rome, 00178 Italy; 4 Azienda Sanitaria Locale, Roma 4, Distretto 4, Via G. Verdi 1, Rignano Flaminio, Rome, 00068 Italy

**Keywords:** Donkey, Goat, Buffalo, Sheep, ncRNAs

## Abstract

**Background:**

Milk is essential for mammalian nutrition because it provides vital nutrients for growth and development. Milk composition, which is influenced by genetic and environmental factors, supports lactation, a complex process crucial for milk production and quality. Recent research has focused on noncoding RNAs, particularly microRNAs (miRNAs), which are present in body fluids and regulate gene expression post-transcriptionally. This study comprehensively characterizes miRNAs in milk of four livestock species, namely *Bubalus bubalis*, *Capra hircus*, *Equus asinus*, and *Ovis aries* and identifies potential target genes.

**Results:**

High-throughput sequencing of milk RNA resulted in distinct read counts across species: *B. bubalis* (8,790,441 reads), *C. hircus* (12,976,275 reads), *E. asinus* (9,385,067 reads), and *O. aries* (7,295,297 reads). *E. asinus* had the highest RNA mapping rate (94.6%) and *O. aries* the lowest (84.8%). A substantially greater proportion of miRNAs over other small RNAs was observed for the donkey milk sample (7.74%) compared to buffalo (0.87%), goat (1.57%), and sheep (1.12%). Shared miRNAs, which included miR-200a, miR-200b, miR-200c, and miR-23a among others, showed varying expression levels across species, confirmed by qPCR analysis. Functional annotation of predicted miRNA target genes highlighted diverse roles, with an enrichment in functions linked to metabolism and immunity. Pathway analysis identified immune response pathways as significant, with several miRNAs targeting specific genes across species, suggesting their regulatory function in milk.

**Conclusions:**

Both conserved and species-specific miRNAs were detected in milk of the investigated species. The identified target genes of these miRNAs have important roles in neonatal development, adaptation, growth, and immune response. Furthermore, they influence milk and meat production traits in livestock.

**Supplementary Information:**

The online version contains supplementary material available at 10.1186/s12864-024-10783-4.

## Background

Milk is a crucial biological fluid for mammals as it serves as the source of energy and nutrients essential for the proper growth and development of living organisms. Milk contains a balanced composition of macronutrients (proteins, lactose, and lipids) and micronutrients (vitamins and minerals), as well as various other bioactive compounds that provide significant benefits during early life stages [1]. The complex composition of milk is a result of the intricate and dynamic process of lactation, which occurs in the mammary gland [2, 3]. This process is influenced by a variety of sources of variation, including genetic, epigenetic, and environmental factors. Proper regulation of lactation is crucial not only to optimize milk production and quality but also to serve as a model for fundamental cellular processes such as proliferation, differentiation, survival, and apoptosis, which can impact milk yield and health outcomes such as mastitis and breast cancer. The amount of data on endocrine regulation and signaling pathways that underly the physiological processes in the mammary gland have increased notably in the last years [4–8].

While protein-coding regions typically represent less than 2% of a mammalian genome, a significant portion of genome is transcribed as noncoding RNAs (ncRNAs) [1], which can be classified based on transcript size into long- and short-RNAs. Emerging evidence indicates that ncRNAs are highly heterogenous RNAs with important regulative roles governing physiology and disease status of the cells [9].

Mature microRNAs (miRNAs) represent a significant class of short ncRNAs of approximately 22 nucleotides in length, first discovered in *Caenorhabditis elegans* in 1993 [10]. These molecules regulate multiple cellular processes through post-transcriptional repression of gene expression. This occurs via binding to the 3’-UTRs of mRNAs, resulting in the inhibition of translation initiation or elongation and the promotion of co-translational protein degradation [11, 12]. MiRNAs play a key role in fine-tuning cellular processes such as modulating animal development, maintaining homeostasis, mediating immune responses, and controlling infections. They are also essential for regulating stem cell self-renewal and tissue differentiation [8] and represent great molecular markers for phylogenetic and taxonomic studies [13]. Upon receiving a physiological stimulus or sustaining an injury, circulating miRNAs (c-miRNAs) can be released from cells into the bloodstream or other body fluids, either actively through secretion or passively through membrane leakage [14–16]. The interest in c-miRNAs stems from their role in regulating molecular pathways in recipient cells and their potential as easily accessible biomarkers for various diseases and disorders [8].

MiRNAs in milk can either be actively secreted by the mammary gland [17] or passively leaked by mammary gland cells [18]. Their expression profiles vary between colostrum and milk [19] and differ among cattle breeds [17]. A comprehensive sequencing analysis of colostrum and raw milk at different lactation stages has identified miRNAs such as miR-181a, miR-155, and miR-223, which are involved in immune response and immune system development and have been found to be significantly more abundant in colostrum than in milk [2–19].

This study aims to provide a comparative characterization of miRNAs in the milk of four economically relevant mammalian species, *B. bubalis*, *C. hircus*, *E. asinus*, and *O. aries*, to gain a thorough understanding of the miRNAs profiles across species. Specifically, the research targets to emphasize the presence of key candidate miRNAs and assess their variability among the four species. The possible most significant overlapping miRNAs could potentially act as biomarkers for specific biological processes and functions.

## Results

### High-throughput sequencing of the Milk miRNAome in four livestock species

**Fig. 1:**
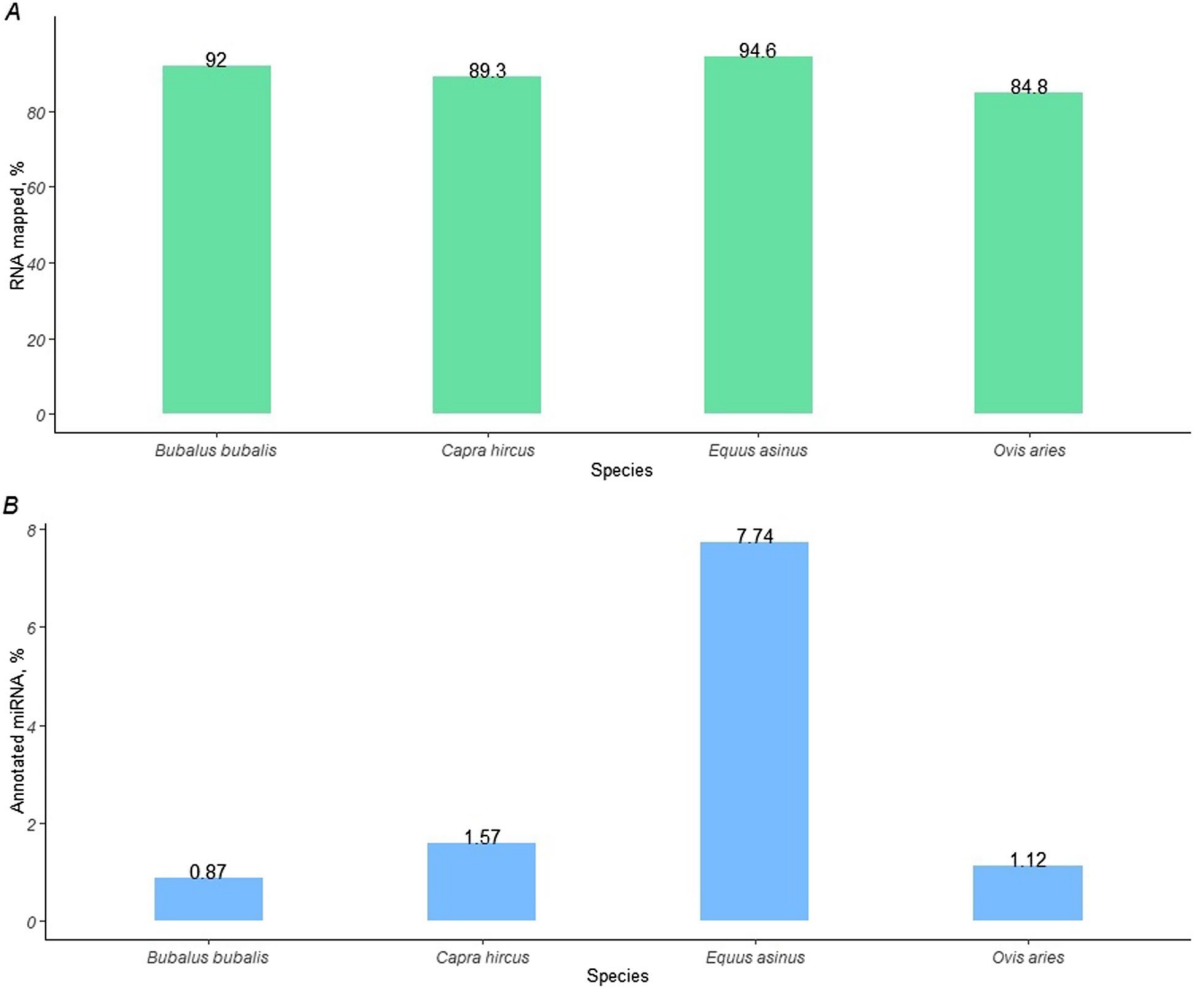
Percentage of the total RNA mapped (**A**) and percentage of the annotated miRNAs (**B**) on *B. bubalis, C. hircus, E. asinus,* and* O. aries* genomes

High-throughput sequencing of milk short RNA yielded 59,974,585 reads for *B. bubalis*, 59,441,932 reads for *C. hircus*, 70,549,149 reads for *E. asinus*, and 57,742,434 reads for *O. aries*. After quality trimming, 8,790,441 RNA reads in milk of *B. bubalis*, 12,976,275 reads in milk of *C. hircus*, 9,385,067 reads in milk of *E. asinus*, and 7,295,297 reads in milk of *O. aries* were retained. The total number of mapped reads is depicted in Fig. 1A, where *E. asinus* had the highest RNA mapping rate (94.6%) and *O. aries* the lowest (84.8%). For *B. bubalis* and *C. hircus*, the percentages of RNA mapped to their reference genomes were 92% and 89.3%, respectively. Of the total annotated RNA for *E. asinus*, 7.74% was identified as miRNAs. The proportions were lower for *B. bubalis*, *C. hircus*, and *O. aries*, with miRNAs representing 0.87%, 1.57%, and 1.12% of total annotated RNA, respectively (Fig. 1B). This highlights the high abundance of miRNAs in donkey milk compared to milk of the other species. Overall, 132 miRNAs in goat, 130 in donkey, 83 in buffalo, and 43 in sheep were identified (Additional file 1).

### Annotation of miRNAs in the four livestock species

**Fig. 2:**
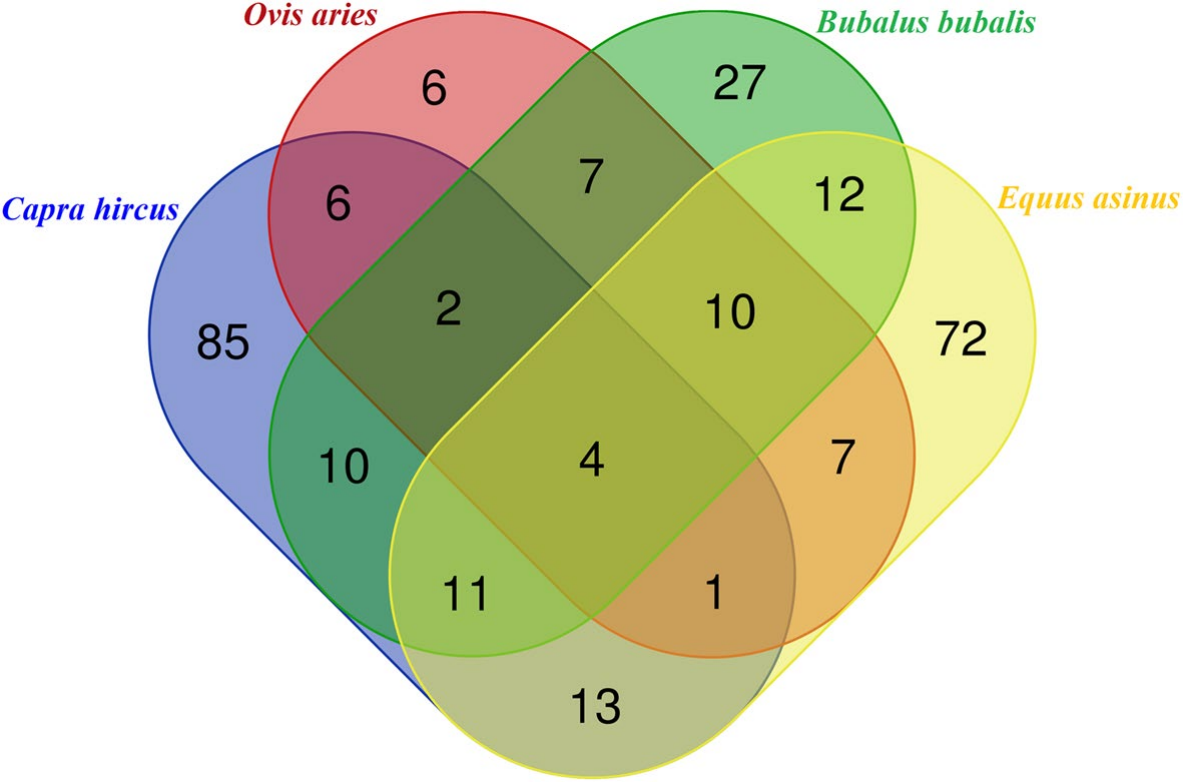
Venn diagrams of the miRNAs shared among the species *B. bubalis, C. hircus, E. asinus,* and* O. aries*

A Venn diagram illustrating the miRNAs shared in the milk of the four livestock species under investigation is reported in Fig. 2. Interestingly, only four miRNAs, namely miR-200a, miR-200b, miR-200c, and miR-23a were shared among all the four species. Furthermore, upon closer examination, several miRNAs were found in milk of multiple species: two in the milk of buffalo, sheep, and goat (miR-30a-5p, miR-22-3p); ten in the milk of goat, donkey, and buffalo (miR-148a, miR-let-7c, miR-29a, miR-let-7 g, miR-30d, miR-let-7f, miR-374a, miR-25, miR-143, miR-221); one in the milk of goat, sheep, and donkey (miR-194); and eleven in the milk of goat, buffalo, and donkey (miR-141, miR-429, miR-423-5p, miR-34a, miR-423-3p, miR-19a, miR-146a, miR-151-5p, miR-660, miR-345-3p, miR-345-5p).

**Fig. 3:**
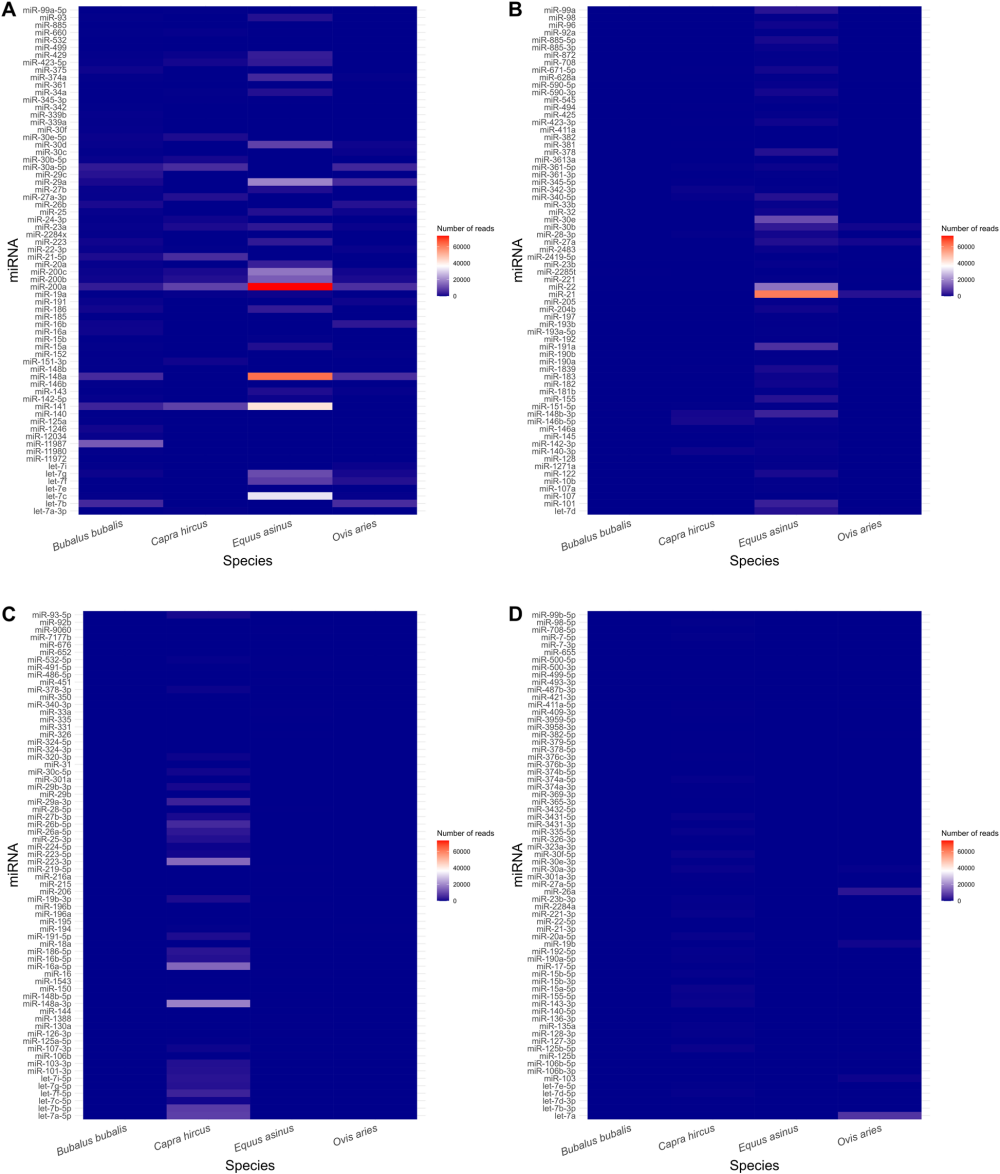
Heatmaps of the shared miRNAs among species. Panels **A**, **B**, **C**, and **D** are represented to make the expression pattern of each miRNAvisible

The heatmaps (Fig. 3A, B, C and D) compares the read counts of all characterized miRNAs in the milk of the four species. It is immediately clear that *E. asinus* had the highest number of miRNAs in milk, followed by *C. hircus*, *B. bubalis*, and *O. aries*. As mentioned earlier, some shared miRNAs had a significantly higher number of reads in one species compared to another. For instance, miR-200a was more abundant in *E. asinus* (15% of the total miRNA reads) compared to *C. hircus* (5%), *O. aries* (11%), and *B. bubalis* (6%). The same trend was observed for the other three shared miRNAs across species, with donkey milk having the highest relative amount. Specifically, for miR-200b, 2% were detected in donkey, 1% in goat, 1% in sheep, and 1% in buffalo milk and for miR-200c, 3% were identified in donkey, 0.7% in goat, 1% in sheep, and 0.7% in buffalo milk. The trend in read counts for miR-23a showed the highest percentage in goat (0.8%), followed by donkey (0.6%), sheep (0.5%), and buffalo milk (0.4%). The number of reads for each identified miRNAs and for each species is reported in Additional file 1.

### Validation of the sequencing data through qpcr

**Fig. 4:**
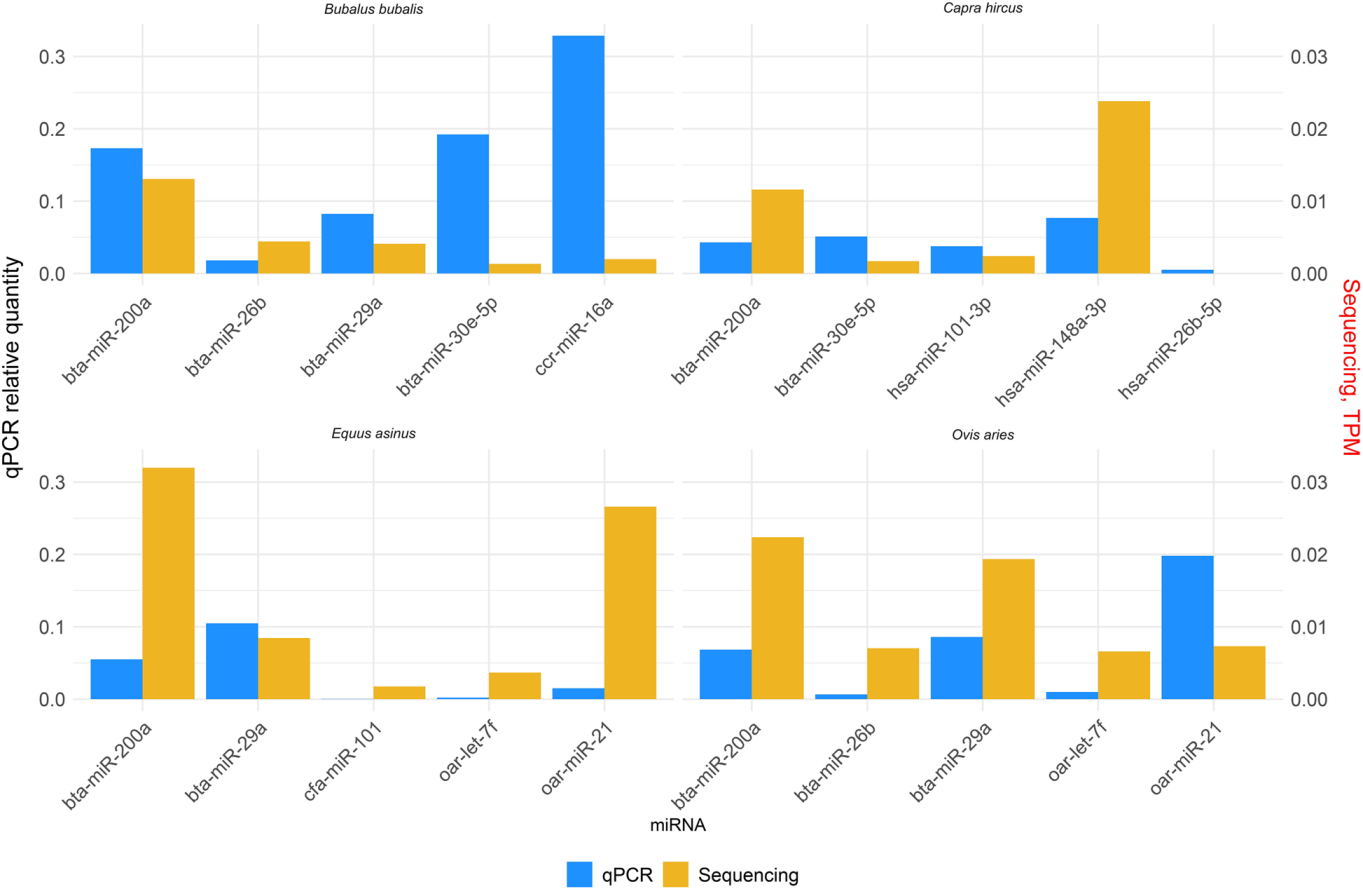
Relative expression of miRNAs analysed through qPCR for each species

The expression levels of some representative miRNAs obtained through RNA sequencing were compared with the results from qPCR obtained through specific primers for the miRNAs. The comparison proved concordance between the up-regulated miRNAs identified in RNA sequencing and their up-regulation in qPCR, as well as a consistent trend for down-regulated miRNAs. Therefore, the qPCR data supported the miRNAs levels observed via RNA sequencing (Fig. 4).

### Characterization of predicted target genes

**Fig. 5:**
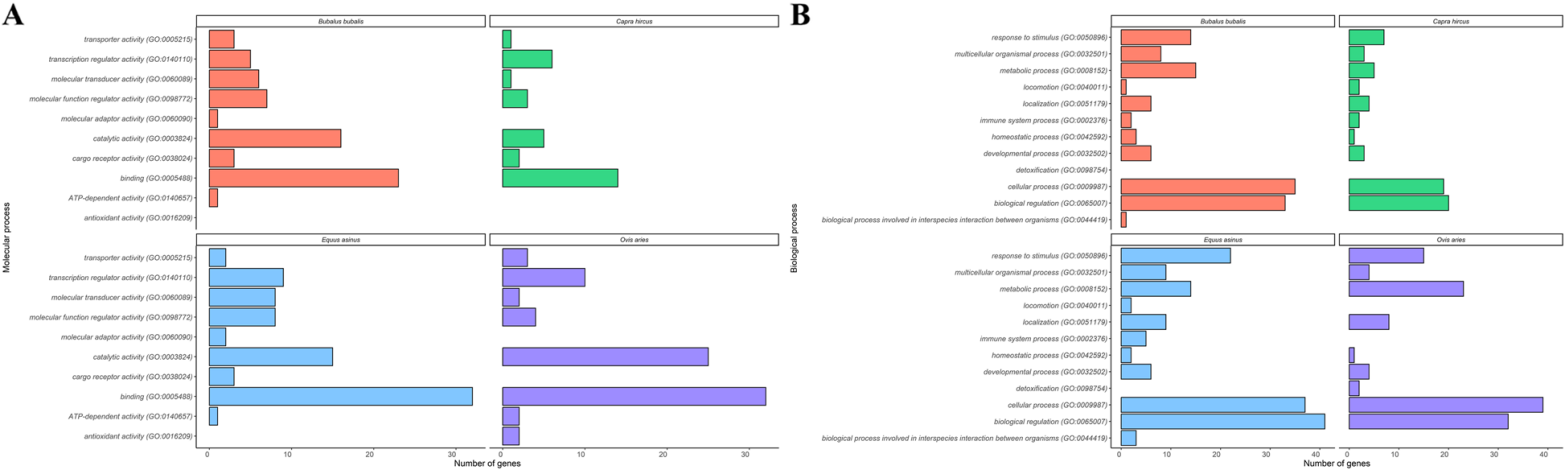
Molecular (**A**) and biological (**B**) processes associated with the miRNA-targeted genes

**Fig. 6:**
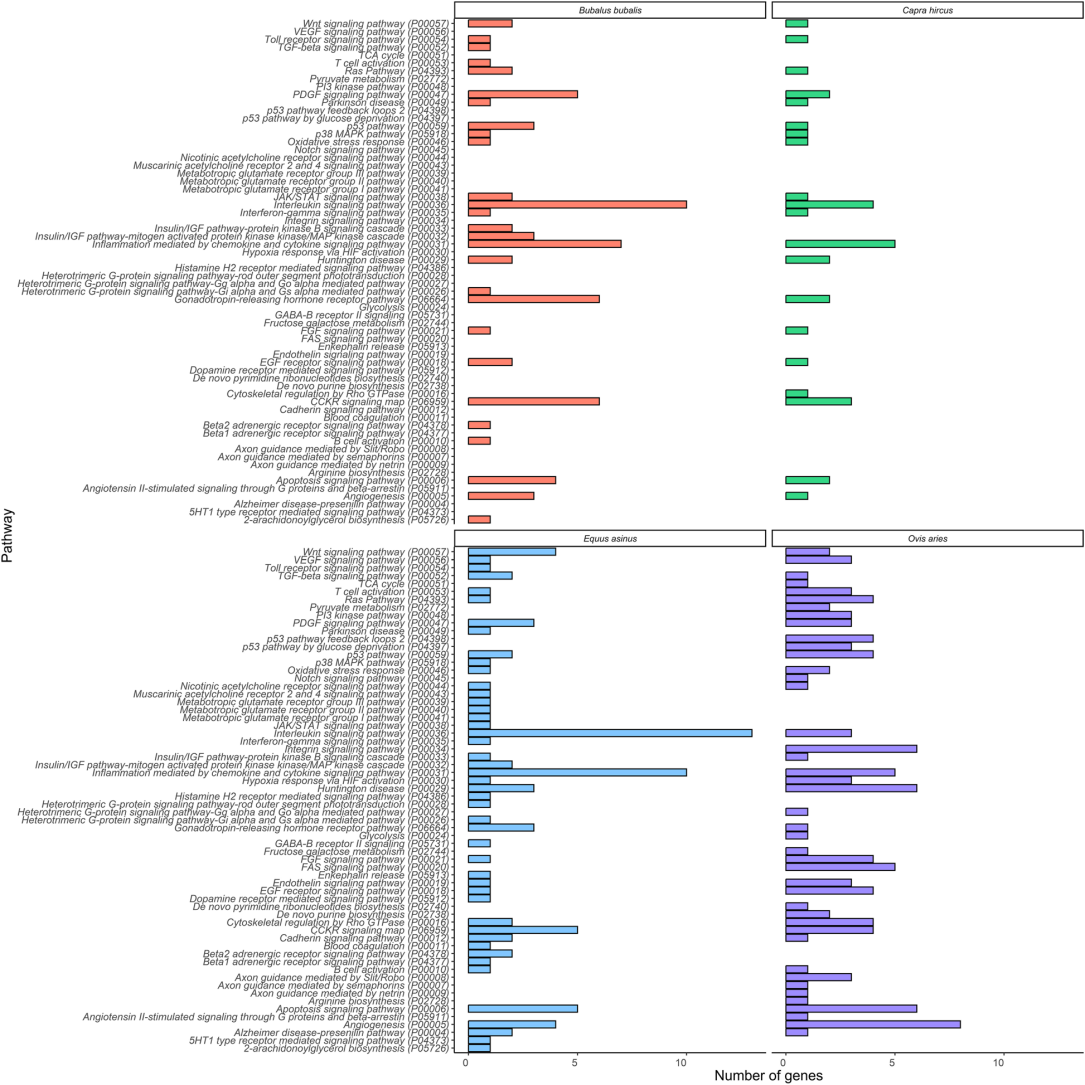
Pathways predicted by KEGG analysis of the miRNA-targeted genes

To delve deeper into the functional annotation of the target genes for each species (Additional file 2), gene ontology (GO) annotation and KEGG analysis were conducted (Additional file 3). The analysis revealed that the targets have a broad spectrum of diverse functions, including involvement in protein and lipid metabolism, tissue development and differentiation, and immune function. Among the molecular processes regulated by these target genes, binding and catalytic activity functions were found particularly significant (Fig. 5A). GO analysis highlighted the participation of the predicted target genes in cellular processes, biological processes, and response to stimuli (Fig. 5B). Upon exploring the cellular pathways in which the predicted target genes of the miRNAs were involved, the most significant pathways were those associated with immune response, such as interleukin signaling and inflammation mediated by chemokine signaling (Fig. 6). The predicted target genes of the miRNAs varied across species and showed variability in terms of number of genes: 121 candidate genes were identified for *B. bubalis*, 55 or *C. hircus*, 135 for *E. asinus*, and 132 for *O. aries*. Among all the predicted target genes identified across species, some were targets of multiple miRNAs and were present among the targets of all the four species. For example, the gene AP2 associated kinase 1 (*AAK1*) was targeted by four miRNAs (miR-221, miR-17-5p, miR-155, miR-205) in three species.

## Discussion

### Characterization of milk miRNAs in four livestock species

In this study we comparatively characterized the miRNAs in milk samples of four livestock species: *B. bubalis*, *C. hircus*, *E. asinus*, and *O. aries*. The composition and abundance of miRNAs in milk can vary significantly between species due to genetic differences, physiological characteristics, and evolutionary divergence. Each species has evolved unique physiological adaptations and metabolic pathways, which can influence the production and secretion of miRNAs into milk. These variations are not only reflective of evolutionary processes but have also practical implications. Understanding species-specific miRNAs profiles can aid in developing targeted strategies for livestock management, breeding, and nutritional interventions. Although miRNAs can be released by flaking cells of the mammary epithelium following mastitis, several studies have demonstrated that miRNAs in milk are also contained in exosomal vesicles released by healthy mammary epithelium [17, 20–22].

Milk of *E. asinus* had a higher relative abundance of miRNAs compared to the milk samples of the other tested species. This finding is consistent with a recent study which has demonstrated elevated miRNAs in donkey milk compared to other species [20]. However, our study did not specifically investigate whether these miRNAs are predominantly present in extracellular vesicles or in free in the matrix, as the focus was on detecting miRNAs in milk, although recent studies have highlighted the importance of exosomes for milk transportation and stability, as well as their intestinal absorption upon milk ingestion [21, 22].

The differential abundance of annotated miRNAs in the milk of various livestock species, particularly the greater quantity observed in donkey compared to sheep, buffalo, and goat milk, raises intriguing questions regarding underlying biological mechanisms. These differences could stem from a combination of genetic, environmental, and physiological factors unique to each species. Genetic variations among species may play a crucial role in determining the repertoire and abundance of miRNAs in milk. As reported in miRBase, there are 1045 mature miRNAs annotated in *B. bubalis*, 436 in *C. hircus*, 694 in *E. asinus*, and 153 in *O. aries*, confirming the genetic variation of miRNAs among these species.

Additionally, environmental factors such as diet, climate, and management practices could influence the expression of miRNAs in mammary glands, ultimately affecting their presence in milk. Moreover, the regulation of miRNAs expression may differ across species due to variations in genetic regulatory mechanisms. The observed differences in miRNAs profiles may also reflect species-specific biological roles. Donkeys, for instance, might possess distinct biological processes or adaptations necessitating a broader range or higher quantity of miRNAs in milk compared to other species [23, 24].

Another interesting result is that milk from donkey, buffalo, sheep, and goat contain only four shared miRNAs (Figs. 2 and 3). Notably, miR-200a and miR-23a are implicated in fatty acid synthesis and have been characterized in the milk of goats, donkeys, and buffaloes but not sheep [25–27]. Moreover, miR-200a is involved in milk production, inflammatory and immune responses [28, 29]. Our results allow speculations on the conservation of certain miRNAs across species, suggesting that the presence of these molecules, while influenced also by diet, may still be maintained to some extent across species. Indeed, miRNAs such as miR-200a and miR-23a are not only present in the milk of the species under investigation but are also conserved in other species such as humans and cattle [30, 31]. Regarding miR-200c, studies have reported its presence in sheep milk and its contribution fat synthesis by targeting the Pantothenate Kinase 3 (*PANK3*) gene [32]. Moreover, it can serve as a biomarker for early pregnancy in cattle [1, 21]. Highly abundant in bovine milk [1, 21, 33], this miRNA has been characterized in sheep milk [32] and donkey milk [34], but not in buffalo and goat milk, so far.

Additionally, miR-200b, belonging to the same miRNAs’ family as the previously mentioned miR-200a and miR-200c, plays a physiological role in mammary tissue development and the lactation ability of mammary epithelial cells in cattle [1–3, 35–38]. Despite its presence among the four species studied, in the literature, it has only been characterized in goat [39], buffalo [40], and sheep milk [27].

MiRNAs, miR-30a-5p, and miR-22-3p, identified in buffalo and goat, seems to be involved in modulating the inflammatory and immune response by influencing the development and differentiation of T lymphocytes, respectively [1, 41]. These two miRNAs are not present in donkey milk, which agrees with findings of the literature [1, 41].

The ten miRNAs shared among goat, donkey, and buffalo were absent in sheep milk, which aligns with existing literature [1, 25, 27, 34]. MiR-148a and miR-25 are involved in fat and lipid metabolism [1, 42], while miR-let-7c serves as a significant onco-suppressor in humans [43], making its presence in milk potentially interesting as it can be ingested through diet. Certain miRNAs such as miR-374a and miR-29a seem to play a role in resistance to heat stress in cattle, suggesting a similar functionality in the species under investigation [44, 45], although miR-29a has a significant role in regulating milk production traits [46].

MiRNAs found in the milk of buffalo, goat, and donkey species include miR-30d, which is involved in regulating milk traits and immune response in buffalo [47, 48]; miR-let-7f, which is a pregnancy biomarker in buffalo [49]; miR-143, which is involved in goat mammary epithelial development [50]; and miR-221, which is associated with mammary epithelial development in cattle [51] and is a pregnancy biomarker in dairy cattle [21]. Among the 10 miRNAs shared across all the four species, miR-let-7 g remains functionally unknown, although it was associated to maintaining endothelial function [52]. Additionally, miRNAs associated with mammary epithelial development may be found in the milk of various species due to natural tissue shedding or the presence of mastitis, which compromises the proper functioning of mammary tissue, making them potential markers of mastitis. Among goat, sheep, and donkey, only miR-194 was present in the milk of all three species, while it was absent in buffalo. Several studies on buffalo support the absence of miR-194 in this species. The function of miR-194 has not been extensively investigated; however, it seems to be involved in the regulation of fetal and muscle development, and lactation in livestock [53].

Regarding the 12 miRNAs shared among goat, buffalo, and donkey species, we found support in the literature about their absence in sheep. Among these, several miRNAs, such as miR-429, miR-19a, and miR-423-5p, seem to play a key role in heat stress resistance in cattle [1, 54, 55]. Additionally, miR-19a is implicated in fatty acid metabolism, similarly to miR-141, miR-423-3p, miR-19a, and miR-34a [56–59]. Among the miRNAs shared among *B. bubalis*, *E. asinus*, and *C. hircus*, miR-151-5p and miR-660 do not yet have a clear role despite being present in the milk of various species. MiRNAs such as miR-345-3p and miR-345-5p are also normally present in *Bos taurus* milk and are thought to be associated with immune response and heat stress resistance [60]. Similarly, miR-146a is associated with immune response, but its central role lies in being a potential biomarker for mastitis, as recently demonstrated in buffalo [61].

Among all the miRNAs identified in the present study, we find some noteworthy due to their high abundance in the milk of the four species and their differences from those previously discussed (Additional file 1). In *B. bubalis*, miR-11987 is interesting because it has been observed to regulate the immune response in cows with subclinical mastitis [62]. Furthermore, in buffalo, miR-let-7b shows very high levels in milk and has also been found in sheep, indicating its involvement in the immune response of both species [24]. Another ncRNA, miR-29c, found only in *B. bubalis*, is associated with resistance to heat stress, heat-induced oxidative stress, and immune response in cows [63, 64]. Its presence in buffalo milk suggests its adaptation to arid climates, allowing buffalo calves, through maternal milk intake, to adapt more quickly to heat stress [63]. Among the most noteworthy and abundant miRNAs in *C. hircus* milk, miR-223-3p, miR-16a-5p, and miR-let-7a-5p stand out. Each of these miRNAs is responsible for different functions: miR-223-3p is involved in the inflammatory response and mammary gland development, miR-16a in fatty acid metabolism, and miR-let7a-5p in fertility [1, 65, 66]. The presence of miR-21 and miR-let-7c in donkey milk is shared with goat, and our results agree with the literature [20]. MiR-21 is associated with the development of the immune and inflammatory response, while miR-let-7c is linked to the lactation stage in both goats and cows [2, 3, 20]. The presence of miR-22 in donkey milk has been documented and our data are consistent with the literature [20]; however, the role of miR-22 remains unknown. For *O. aries*, we identified a high quantity of miR-let-7a, miR-16b, and miR-7b, the last one shared with the buffalo species and discussed above. MiR-let-7a has never been documented in sheep milk before this study and seems to be associated with the hair follicle growth process [67], while miR-16b is negatively correlated with milk production and proteins in sheep milk [68]. The presence of miR-let-7a in sheep milk, given its function, suggests that its intake through lamb diet may favor the development of the classic wooly phenotype of sheep.

The differences in digestive physiology between monogastric and polygastric animals could significantly influence the presence and composition of miRNAs in milk. Monogastric animals, such as donkeys, have a single-chambered stomach, which processes nutrients differently compared to polygastric animals, such as buffalo, goat, and sheep, which have multi-chambered stomachs designed for fermentative digestion.

In polygastric animals, the complex stomach structure, including the rumen, reticulum, omasum, and abomasum, enables extensive microbial fermentation before nutrient absorption occurs. This process can modify the expression profiles of miRNAs, as it affects the metabolic environment and the bioavailability of miRNAs precursors and other regulatory molecules [19, 28, 30]. For instance, the presence of the rumen microbiome and its fermentative activity might influence the stability and processing of dietary miRNAs, potentially altering their subsequent secretion into milk [38].

Conversely, in monogastric animals, nutrient absorption occurs primarily in the small intestine following enzymatic digestion in a single stomach compartment. This simpler digestive process might lead to different miRNAs profiles in milk, as it involves distinct regulatory pathways for miRNAs synthesis and secretion [38]. Additionally, the less extensive microbial activity in monogastric digestion compared to polygastric fermentation may result in less degradation or modification of miRNAs before they are secreted into the milk [38]. These physiological differences highlight the need to consider the type of digestive system when studying miRNAs profiles in milk from different species. Understanding how these digestive processes influence miRNAs presence can provide deeper insights into the functional roles of these molecules in milk and their potential impacts on neonatal development and health.

### Functional analysis and target gene prediction of milk miRNAs

MiRNAs play crucial regulatory roles by targeting various mRNAs [12]. In order to elucidate the biological functions of miRNAs and identify their putative target genes, we used two algorithms: TargetScan and MiRanda. Specifically, a total of 439 genes were identified among the species, some of which were targeted by 10 miRNAs, while others were targeted by a single miRNA. For example, the gene Tumor necrosis factor (*TNF*) was targeted by 13 miRNAs in all the species under investigation, whereas miR-17-5p targeted 27 genes (Additional file 2).

The GO annotation and KEGG pathway analyses were performed to enhance the understanding of miRNAs functions and to elucidate miRNAs gene regulatory networks. Figure 5A and B depict the molecular and biological processes in which these genes are involved, categorized by species. Notably, our results demonstrate a consistent trend across all species; indeed, the catalytic activity (GO:0009824), binding activity (GO:0005488), cellular processes (GO:0009987), and biological regulation (GO:0065007) are the biological and molecular processes in which the targeted genes are involved, suggesting that miRNAs regulate well-defined processes that are conserved across species [69].

Additionally, the study of pathways of the targeted genes revealed a consistent trend across species (Fig. 5). Most of the targeted genes belong to pathways associated with immune response, such as the interleukin signaling pathway (P000036) and inflammation mediated by chemokine signaling pathway (P00031). Furthermore, there is involvement of candidate genes in the Cholecystokinin receptor (*CCKR*) signaling pathway, which seems to be crucial for nutrient digestion and absorption, as well as appetite and metabolism regulation [70]. The KEGG analysis presented in Additional file 3 seems to confirm the obtained results. Additionally, KEGG analysis showed several targeted genes involved in different pathways and many involved in the same pathway, indicating the functional complexity of miRNAs action.

Among all identified genes considered as potential targets, some stand out for their function. For instance, the gene *AAK1*, targeted by miR-221, miR-17-5p, miR-155, and miR-205, is involved in the immune response and implicated in the development of diseases such as Alzheimer and Parkinson in humans [71]. We also find B-cell translocation gene 2 (*BTG2*), associated with cell differentiation and growth; Cluster of Differentiation 96 (*CD96*), responsible for immune response; and Interleukin 10 (*IL10*), 18, and 6, actively involved in immune response and expression of inflammation-related genes [72–74]. Two other genes strongly implicated in the immune response are *TNF* and TNF Alpha Induced Protein 8 (*TNFAIP8*) [64, 75]. The identification of these genes aligns with the functions of the identified miRNAs. It can be inferred that the absorption of miRNAs through milk consumption may play an important role in the development and enhancement of the immune response.

Another notable gene is AT-Rich Interaction Domain 3A (*ARID3A*), responsible for adaptation to arid climates in sheep and goats, and found to be targeted in our study by miR-146a and miR-let-7i-5p [76], while the gene Fibroblast Growth Factor 7 (*FGF7*) is significantly associated with growth traits in goats [77]. This confirms the importance of miRNAs in milk, particularly concerning the health, adaptability, growth, and immune defense of calves, kids, foals, and lambs.

However, we believe that there are further noteworthy candidate genes, especially those linked to livestock production traits. For example, the gene Acyl-CoA Synthetase Long Chain Family Member 5 (*ACSL5*) in buffalo is associated with milk production traits [78], while the gene Butyrophilin Subfamily 1 Member A1 (*BTN1A1*) is associated with milk traits in dairy goats [79]. Genes such as Glycerol-3-Phosphate Acyltransferase—Mitochondrial (*GPAM*), Heat Shock Protein Family A Member 8 (*HSPA8*), Low-Density Lipoprotein Receptor (*LDLR*), and Suppressor of Cytokine Signaling 3 (*SOCS3*) are associated with milk components, as they are responsible for triglyceride metabolism, milk protein concentration, and cholesterol content, respectively [80–83]. Finally, the target genes of miRNAs such as Adrenergic Receptor Beta 2 (*ADRB2*), Arrestin Domain Containing 4 (*ARRDC4*), and Cathepsin C (*CTSC*) are responsible for meat traits and carcass quality [84–86].

## Conclusion

We observed that the characterized miRNAs in milk are conserved across all species, while others are specific to certain species, similarly to findings from the literature. Additionally, we evaluated which genes are targeted by the miRNAs present in milk, revealing their crucial roles in the development, adaptation, growth, and immune response of newborns. Moreover, these miRNAs can modulate the production traits in livestock. We believe that future studies should focus on defining these targets in milk from individual animals, considering lactation stage, age, and parity order to deepen their presence in milk. Our study provided a general but comprehensive overview of the miRNAs community in the milk of four species.

## Methods

### Sampling, RNA extraction, and sequencing

Individual milk samples were collected in Italian commercial farms of *B. bubalis*, *C. hircus*, *E. asinus*, and *O. aries* using mobile electronic milk meter (LactoCorder®, WMB, Balgach) by the personnel of the “Istituto Zooprofilattico Sperimentale del Lazio e della Toscana “M. Aleandri”—National Reference Centre for Ovine and Caprine Milk and Dairy Products Quality” (Rome, Italy) during routine mechanical milking procedures. All the farms were located in the Lazio region (Italy). For *E. asinus*, 49 females of the Amiata (*n* = 37) and Ragusana (*n* = 12) breeds from parity 1 to 7 and 2 to 7 months in lactation were sampled in two farms. For *C. hircus*, 498 lactating goats from the Alpine (*n* = 194), Saanen (*n* = 186), and Maltese (n = 68) breeds, and crossbreeds (*n* = 50) from parity 1 to 5 and 1 to 7 months in lactation were sampled in five farms. For *O. aries*, 443 animals from the Assaf (*n* = 28), Comisana (*n* = 81), Lacaune (*n* = 36), Sarda (*n* = 267), and Sopravissana (*n* = 31) breeds from parity 1 to 6 and 1 to 7 months in lactation were sampled in five farms. For *B. bubalis*, 648 individuals of the Italian Mediterranean buffalo from parity 1 to 7 and 1 to 11 months in lactation were collected in five farms. The sampling process described above led to 20, 32, 13, and 20 pooled samples for *E. asinus*, *C. hircus*, *O. aries*, and *B. bubalis*, respectively.

The buffaloes were fed a diet consisting of unifeed supplemented with hay and feed, while the goats, sheep, and donkeys grazed freely, receiving additional supplementation of hay and grains. The pooled samples were immediately placed on ice after collection in the herd, transported to the laboratory of the Department of Agronomy, Food, Natural resources, Animals and Environment of the University of Padova (Italy), and stored at -80 °C until the end of the sampling process which lasted one year. Subsequently, all samples were allowed to thaw overnight at room temperature, and they were thoroughly mixed to ensure uniformity. This procedure prevented the formation of layers, such as the fat layer or skim milk, which could potentially introduce biases in miRNAs characterization. Indeed, focusing on specific layers could lead to errors in miRNAs detection, as certain miRNAs may localize in the fat layer or precipitate with proteins.

To maximize variability, a single pool (50 ml) of milk for each species was prepared by combining equal proportions from all pooled sampled herds for total RNA extraction. The approach of using pooled samples aligns with established methodologies in the field of miRNAs research [87, 88]. Pooling samples allows for a more comprehensive assessment of overall miRNAs expression profiles, capturing biological variability across multiple samples. This approach is particularly useful for studies aimed at exploring and characterizing miRNAs in complex matrices like milk. Previous studies have often used much smaller sample sizes than our research, which may have introduced potential biases due to the inability to account for all factors affecting the presence or absence of miRNAs. Our study addresses this limitation by utilizing a significantly larger sample size compared to previous literature [17, 87, 89, 90]. While random pooling rather than meticulous balancing of different factors might result in some under- or over-representation, we are confident that the large sample size has effectively captured a broad range of conditions (e.g., management practices, feeding regimes, physiological status), and thus it has ensured the representativeness and robustness of data, and a comprehensive representation of miRNAs in each species [91].

The RNA was isolated from 2 ml of the matrix using the exoRNeasy Maxi kit (Qiagen, Venlo, The Netherlands) according to the manufacturer's instructions, with an elution volume of 14 µl. Library preparation was carried out using the QIAseq miRNA Library Kit (Qiagen, Venlo, The Netherlands). A total of 100 ng of RNA was used to prepare the miRNA NGS libraries. After adapter ligation, Unique Molecular Identifiers (UMIs) were introduced during reverse transcription. The cDNA was amplified using PCR (16 cycles) and the products were purified. Library preparation quality was controlled using capillary electrophoresis (Agilent Tape D1000, Santa Clara, US). Based on insert quality and concentration measurements, the libraries were pooled in equimolar ratios. The library pools were quantified using qPCR and subsequently sequenced on a NextSeq (Illumina Inc., San Diego, California, US) instrument according to the manufacturer's instructions with a 75 bp single-end read layout. Raw data were de-multiplexed and FASTQ files for each sample were generated using the bcl2fastq2 software (Illumina Inc., San Diego, California, US).

### Data analysis

Short non-coding RNA reads in FASTQ format were trimmed using cutadapt v. 1.18 [92] to remove adapter sequences and bases with a PHRED score below 25. After quality trimming, the reads were selected based on a size range of 18 to 35 nt. The trimmed reads were analysed using miRTrace v.1.0.0 [93] to cluster similar sequences and assess the dataset quality, size distribution, and potential contaminants such as xenomirs (miRNAs of different lineages). The trimmed reads were mapped to the *B. taurus* (NCBI genome, ARS-UCD1.2—GCA_002263795.2), *C. hircus* (NCBI genome, ARS1.2 – GeneBank ID: GCA_001704415.2), *E. caballus* (NCBI genome, EquCab3.0 – GeneBank ID: GCA_002863925.1), and *O. aries* genomes (NCBI genome, ARS-UI_Ramb_v3.0 – GeneBank ID: GCA_016772045.2), using the CLC mapper (CLC Genomics, Qiagen, Venlo, The Netherlands) with a similarity threshold of 0.9 across the entire read length. Both the total number of mapped reads and reads mapped to each genomic feature type (coding genes, rRNAs, miRNAs) were counted and visualized.

To identify bona fide miRNAs, the following annotation criteria were applied: (i) presence of coverage on both arms of the miRNA sequences, (ii) a distance between mature and star sequences less than 40 nt, (iii) absence of mapped reads in the vicinity of the annotated miRNAs, (iv) 5′ homogeneity of the mature miRNAs, and (v) low free energy. The genomic locus of each bona fide miRNA was determined using blastn, while miRNA identities were confirmed using blastn against the miRBase [94] and MirGeneDB databases [95]. The expression levels of miRNAs were calculated as the number of mapped reads normalized by the total number of mapped reads (reads per million of mapped reads, RPKM).

Differentially expressed miRNAs were identified and their predicted target genes were estimated using miRanda and TargetScan v7.0 software [96]. The sequences of the genomes used for target prediction analysis were downloaded from NCBI database, considering the last annotations. For the TargetScan v7.0, a seed match = 7 and context + score percentile = 99 was settled as the threshold, and for the miRanda a free energy = -20 (kcal/mol) was chosen as a cutoff. The intersection of these prediction results was taken as the set of candidate target genes [96]. The GO and KEGG analyses of predicted target genes were estimated using the PantherGO (https://www.pantherdb.org/) [97] and Database for Annotation, Visualization and Integrated Discovery (DAVID) (https://david.ncifcrf.gov/) [98], respectively.

### Validation of milk miRNAs by qPCR

The expression levels of selected miRNAs for each species were evaluated using qPCR with the miRCURY LNA miRNA SYBR Green PCR kit (Qiagen, Venlo, The Netherlands). When possible, LNA probes were sourced from catalog products; otherwise, they were designed using the GeneGlobe platform (https://geneglobe.qiagen.com/). First-strand cDNA was synthesized from 50 ng of total RNA using the miRCURY LNA RT Kit (Qiagen, Venlo, The Netherlands), following the manufacturer's protocol with the following cycle: 60 min at 42 °C, 5 min at 95 °C, and immediate cooling to 4 °C. The resulting cDNA was subjected to five dilutions, ranging from 1:10 to 1:200, and used to assess probe efficiency in an initial qPCR plate. All designed probes showed high efficiency (R^2^ > 0.95) across sequential cDNA dilutions. This was determined by calculating the R^2^ values of the standard curves and using the formula: E = 10^(-1/slope)—1. All probes used in the present study were purchased from Qiagen and are listed on their website (https://www.qiagen.com/us) with the following IDs: YP02104134 (bta-miR-200a), YP00205953 (bta-miR-26b), YP02114732 (bta-miR-29a), YP02118996 (bta-miR-30e-5p), YP02101072 (ccr-miR-16a), YP00204786 (has-miR-101-3p), YP00205867 (has-miR-148a-3p), YP00204172 (has-miR-26b-5p), YP00205955 (cfa-miR-101), YP02106634 (oar-let-7f), YP02110192 (oar-miR-21) and YP00203954 (UniSp6).

Final qPCR reactions were conducted with 3 μl of 1:100 cDNA in a 10 μl reaction mixture (5 μl of 2X Master Mix, 0.5 μl of Rox passive reference dye, 1 μl of probes, and 0.5 μl of water). Amplification cycles were performed on an AriaMx Real-Time PCR System (Agilent Technologies Inc., Santa Clara, US) with the following parameters: 95°C for 2 min followed by 40 cycles at 95°C for 10 s and 56°C for 1 min. Subsequently, a dissociation curve analysis was conducted to confirm probe specificity. Each qPCR assay was performed in triplicate on the same plate for each probe. To determine relative expression ratios, the internal control Unisp6-spike (Qiagen, Venlo, The Netherlands) was used. The relative expression level was calculated using the comparative 2^–ΔΔCt^ method [99].

Several studies have validated the use of pooled samples for sequencing and qPCR [24, 87, 88]. Using the same pools for both sequencing and qPCR aimed to confirm sequencing results and minimize false positives.

## Supplementary Information

Supplementary Material 1.

Supplementary Material 2.

Supplementary Material 3.

## Data Availability

The data presented in the study are deposited in the Sequence Read Archive (NCBI—SRA) repository (https://www.ncbi.nlm.nih.gov/bioproject/PRJNA1130716), under accession number: PRJNA1130716.
